# Topical Application of Activity-based Probes for Visualization of Brain Tumor Tissue

**DOI:** 10.1371/journal.pone.0033060

**Published:** 2012-03-13

**Authors:** Jennifer L. Cutter, Nathan T. Cohen, Jing Wang, Andrew E. Sloan, Alan R. Cohen, Ashok Panneerselvam, Mark Schluchter, Galia Blum, Matthew Bogyo, James P. Basilion

**Affiliations:** 1 Department of Radiology, National Foundation for Cancer Research Center for Molecular Imaging at Case Western Reserve University, Cleveland, Ohio, United States of America; 2 Department of Biomedical Engineering, National Foundation for Cancer Research Center for Molecular Imaging at Case Western Reserve University, Cleveland, Ohio, United States of America; 3 Department of Neurological Surgery, Stanford University School of Medicine, Stanford, California, United States of America; 4 Pediatric Neurological Surgery, University Hospitals Case Medical Center, Stanford University School of Medicine, Stanford, California, United States of America; 5 Department of Epidemiology and Biostatistics, Stanford University School of Medicine, Stanford, California, United States of America; 6 Department of Pathology, Stanford University School of Medicine, Stanford, California, United States of America; 7 Department of Microbiology and Immunology, Stanford University School of Medicine, Stanford, California, United States of America; University of Texas - M.D. Anderson Cancer Center, United States of America

## Abstract

Several investigators have shown the utility of systemically delivered optical imaging probes to image tumors in small animal models of cancer. Here we demonstrate an innovative method for imaging tumors and tumor margins during surgery. Specifically, we show that optical imaging probes topically applied to tumors and surrounding normal tissue rapidly differentiate between tissues. In contrast to systemic delivery of optical imaging probes which label tumors uniformly over time, topical probe application results in rapid and robust probe activation that is detectable as early as 5 minutes following application. Importantly, labeling is primarily associated with peri-tumor spaces. This methodology provides a means for rapid visualization of tumor and potentially infiltrating tumor cells and has potential applications for directed surgical excision of tumor tissues. Furthermore, this technology could find use in surgical resections for any tumors having differential regulation of cysteine cathepsin activity.

## Introduction

In 2008, an estimated 21,000 men and women were diagnosed with cancer of the brain [1]. Of these, glioblastoma multiforme (GBM) is the most common, accounting for 31% of all tumors and 80% of malignant brain tumors (CBTRUS, 2011, http://www.cbtrus.org/2011-NPCR-SEER/WEB-0407-Report-3-3-2011.pdf). These types of tumors present as focal masses with margins infiltrating the brain parenchyma. The current treatment for malignant brain tumors includes surgical excision (if possible) of the neoplastic mass followed by radiation of the resection cavity and often adjuvant chemotherapy to help prevent recurrence. However, despite decades of research efforts these measures have only minimally increased the average lifespan of patients [2]. Therefore the development of novel approaches that can be used to refine current approaches for diagnosis and treatment of these cancers remains imperative.

Several technological advances have been developed which improve the quality and efficacy of brain tumor surgery. These include microscopic and MRI enhanced surgery and intraoperative fluorescence-guided surgery, although the latter is not yet clinically available in the United States [3], [4]. In 1997 Black introduced MRI based intraoperative imaging (IOI) as an improvement over microscopic surgery alone [5] and in 1999 Knauth et al. demonstrated that IOI MRI was effective at decreasing tumor burden [6], [7], [8], [9]. More recently, clinical trials in Europe have begun to use fluorescence guided surgical techniques to achieve more complete tumor resections [3]. Stummer et al. has demonstrated that 5-ALA fluorescence-guided resection of GBM results in statistically significant increase in complete resections of the tumor, as judged by post-operative MRI, compared to white light resected patients which was correlated with an increase in 6-month progression free survival [3]. This study did not show a long-term increase in survival, but was not powered to do so. Interestingly, advances in neurosurgical techniques have proven to be the most effective method of altering the natural progression of brain cancer, even compared to advances in chemotherapy. A number of studies show a significant correlation between improved resection efficacy and increased patient survival and better quality of life [10] [9], [11], [12]. However, these MRI/fluorescence guided “complete resections” obviously are not removing all the tumor tissue and do not affect cures. With current “complete resections” (MRI negative images post-operatively) there is a statistically significant increase of approximately 5.1 months in patient survival but cures are not achieved [10]. This surgical failure is partially related to probe bioavailability and image resolution, both of which limit the ability of current imaging techniques to accurately define tumor margins and determine the extent of infiltrating cells during surgery. Further, invasive tumor tissues, even if visible, are sometimes not possible to remove without causing significant patient deficits. Therefore, the goal for surgical resection of GBM likely will be to achieve maximal de-bulking of the tumor resulting in extended patient survival [10] thereby potentially increasing the efficacy of adjuvant therapies. A large EORTC study showed that patients with complete resection benefit most strongly from concomitant radiotherapy with temozolomide [13], thus giving a further incentive for maximum resection. To advance the efficacy of brain tumor resection, it will be necessary to clearly identify and remove margin-penetrating cells. Intraoperative microscopic techniques combined with *molecular imaging probes* specific for tumor markers could play a significant role in future surgical and therapeutic approaches. Here we present studies using molecular imaging that exploit tumor associated proteases as markers for identification of tumor tissues.

Upregulation of proteases in cancers is a well documented phenomenon [14]. Several families of cysteine proteases are consistently over-expressed in many types of cancers [15], [16]. In particular, the papain family cysteine proteases cathepsins B and L exhibit increased activity in most cancers [17], have significantly increased expression and activity in GBMs [18], [19], [20], and have the highest degree of expression at the tumor - brain interface [21], [22].

Several investigators have successfully developed fluorescent molecular imaging probes to image disease-associated proteases [23], [24], [25], [26]. More recently these imaging technologies have been further developed to enable image-guided resections of tumors [27], [28], [29]. Most of the non-invasive NIRF imaging probes developed to date are fluorescently quenched substrate based probes and rely on a consensus peptide sequence to serve as an enzyme substrate allowing for probe activation. Here we employ fluorescently quenched activity based probes (ABP) that are based on small molecule suicide inhibitors of tumor-associated proteases. Upon interaction of the probe with its enzyme target it undergoes a bond rearrangement resulting in the loss of the quenching moiety (probe activation) and covalent modification of the active-site cysteine of the protease causing enzyme inhibition and “linking” of the fluorochrome to its target [23]. The stability of the covalent modification of the targeted enzyme presumably keeps the fluorochrome from washing away from the site where it was unquenched making these probes as effective as substrate-based probes, which take advantage of enzymatic activity to amplify the signal by activating more than one probe, but can have significant washout of liberated fluorochromes [30]. Additional benefits of the covalent ABP probes are that they provide higher protease selectivity than do substrate probes and allow direct SDS-PAGE evaluation of labeled targets [23], [31]. Furthermore, these probes rapidly penetrate cells and become activated both *in vitro* and *in vivo* [23], [31].

We present the use of fluorescently quenched activity based probes as novel intra-operative molecular imaging tools for identification of tumor margins and infiltrating tumor cells. Specifically, we have demonstrated that quenched ABPs can be formulated for topical administration, that topically administered probe is activated within minutes by specific cathepsin proteases and that topical administration highlights tumor margins and small clumps of tumor cells as compared to systemically administered probes. Finally, we demonstrate that human GBM biopsies also rapidly activate the probe supporting the argument that this technology has the potential to improve surgical resection of brain tumors. We envision that this technology will provide significant value in the improvement of tissue-sparing procedures, such as brain tumor resections.

## Results

**Figure 1:**
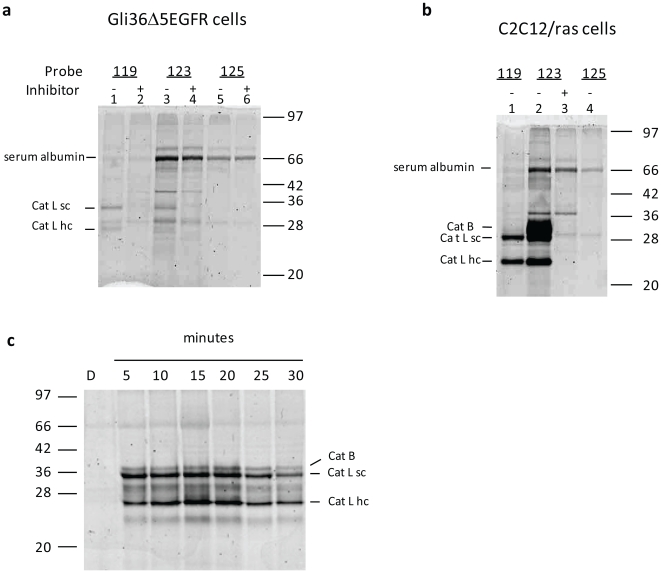
GB119 specifically labels cells *in vitro* and tumor tissue *ex vivo*. (**a**) Gli36Δ5EGFR cells were incubated with JPM-OEt, a general papain-family inhibitor (+, lanes 2,4 and 6) or 0.1% DMSO vehicle control (−, lanes 1,3 and 5) before the addition of the indicated probe GB119, GB123, or GB125 (the control probe, GB125, contains the Cy5 fluorescence label with a non-reactive amide in place of the acyloxymethylketone (AOMK) “warhead”(22)). (**b**) *In vitro* cell labeling with NIRF-APBs. C2C12/ras cells were incubated with JPM-OEt, a general papain-family inhibitor (+) or 0.1% DMSO as a vehicle control (−) before the addition of the indicated probe. Samples were analyzed by SDS-PAGE and Cy5 fluorescence was measured by scanning the gels with a Typhoon scanner. Specific bands are indicated as follows: cathepsin L single-chain form (Cat L sc); cathepsin L heavy-chain (Cat L hc), and cathepsin B (Cat B). (**c**) Gli36D5EGFR flank tumor tissue was immersed in GB119 for the indicated time or DMSO vehicle control (D) for 30 minutes. Specific cathepsin bands are indicated as follows: cathepsin L single-chain form (Cat L sc,); cathepsin L heavy-chain (Cat L hc); and cathepsin B (Cat B).

In order to establish a brain tumor model system that would be suitable for imaging using the ABP probes, we evaluated levels of active cathepsins in the human brain tumor cell line Gli36Δ5EGFR relative to a previously analyzed mouse cell line known to exhibit high cysteine cathepsin levels (C2C12/Hras1) (22). Labeling of intact cells with either the quenched activity based probe (qABP) GB119 or the non-quenched version of the probe, GB123, followed by SDS-PAGE and Typhoon scanning (22) indicated that GB123 labeled both human and mouse cathepsin L (Cat L) and cathepsin B (Cat B), as reported previously for other cells (22). GB119, however, was much more selective for Cat L with little to no labeling of Cat B in mouse or human tissue culture cells. Selectivity of labeling was further confirmed by observing the loss of labeling in cells pre-incubated with a broad-spectrum cathepsin inhibitor, JPM-OEt, (**Fig**. 1 a–b). An additional 66 kD protein which was labeled strongly with GB123 but only weakly with GB119, was identified via mass spectrometry as a non-specific interaction of the probe with serum albumin (data not shown). The control probe, GB125, which contains the Cy5 fluorescence label with a non-reactive amide in place of the acyloxymethylketone (AOMK) “warhead”(22), also strongly labeled this band further supporting the non-specific nature of the interaction. This background signal is reduced in the samples treated with GB119 because albumin cannot activate the quenched probe.

We next determined if GB119 could be activated by cysteine cathepsins within a solid tumor following topical application *ex vivo*. Tumors derived from Gli36Δ5EGFR cells were excised, surgically divided, bathed in 10 µM GB119 or vehicle control and analyzed biochemically as above (**Fig**. 1c). Consistent with the *in vitro* cell labeling data, the probe penetrated the tissue and efficiently labeled both the single and heavy chain forms of Cat L, with less labeling on Cat B. The signal appeared to plateau at 20 minutes with a decrease in signal strength at 25 and 30 minutes, perhaps due to the lack of extended viability of the tissue in this *ex vivo* setting.

**Figure 2:**
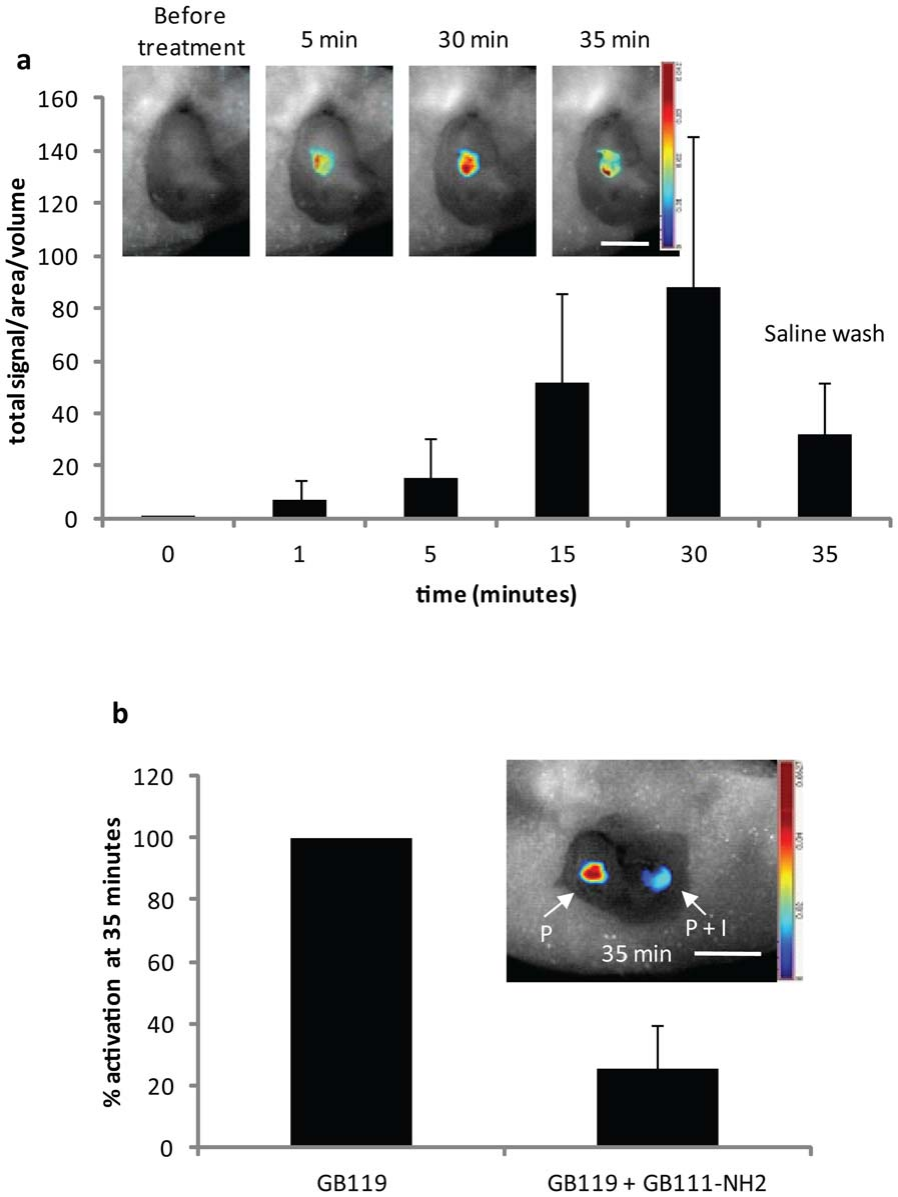
Topical application of GB119 to heterotopic brain tumors results in rapid and specific *in vivo* activation of the probe. (a) Mice bearing Gli36Δ5EGFR flank tumors (n = 12) were treated with GB119 and imaged over time. The mean +/− standard deviation is plotted vs. time. Wilcoxon signed ranked test showed that the signal increased significantly above the time 0 signal at each of the times 1, 5, 15, 30, and 35 (p = 0.0005 at each time point). Inset is a representative mouse treated topically with GB119 and imaged across time. (b) Percent inhibition of GB119 activity by GB111-NH_2_ was determined for each mouse (n = 4) and is presented as mean +/− standard deviation at 35 minutes. There was significant reduction in the signals when incubated with both GB119 and inhibitor at all time points except time 0 (two-sample t-test p<0.05). Inset represents GB119 (P) or a mixture of GB119 with 100-fold excess of GB111-NH_2_ (P+I) at 35 minutes after treatment. Images are unmixed false colored maps of pixel intensity representing activated GB119.

We next sought to test the utility of topical application to specifically identify tumor proteases *in vivo*. Gli36Δ5EGFR cells were used to form flank tumors in nude mice. When tumors were 10–12 days old GB119 was topically applied to the exposed tumor surface *in vivo* and its activation was monitored by fluorescence imaging (**Fig. 2a**). GB119 was rapidly activated with signal detected in as little as 1 minute with continued accumulation up to 30 minutes. Washing and re-imaging of the region of initial probe application decreased signal by nearly 60%, presumably due to removal of secreted labeled-proteases. However, the remaining signal remained statistically significant relative to the control (p-value = 0.0005). To test the *in vivo* specificity we topically treated tissues with either GB119 alone (P) or a mixture of GB119 and GB111-NH_2_, an inhibitor of Cat L and B [23], at 100-fold excess of (P+I) (**Fig. 2b**). In these studies small pieces of tumor were surgically removed to create cavities in the tumor to ensure that the applied treatments were unable to inadvertently combine (inset, **Fig. 2b**). Animals were imaged every 5 minutes for 30 minutes at which time the cavities were washed and re-imaged at 35 minutes. Inclusion of the inhibitor during incubation reduced probe activation by 74.5%. To better understand the specificity of the tissue labeling, GB119 was topically added to flank tumors *in vivo* and the resulting fluorescing tissue was removed and subjected to SDS-PAGE analysis. These studies showed that *in vivo* GB119 retained its specificity to label Cat L (**figure S1**). Further studies with a broad spectrum cathepsin inhibitor, JPM-OEt, also confirmed that *in vivo* activation of the probe was protease specific (data not shown). As expected, similar topical *in vivo* studies using non-quenched ABPs, such as GB123, suffered from high background levels, which could not be removed, and tumor tissue could, therefore, not be resolved from background.

**Figure 3:**
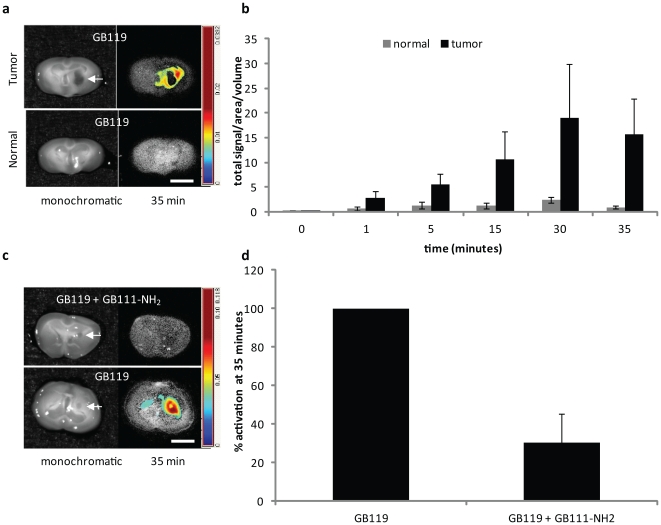
*Ex vivo* activation of GB119 in orthotopic brain tumors. (**a**) Representative 2-mm sections from normal and tumor-bearing brains (see arrow for main tumor mass). Monochromatic images are shown on left and unmixed false colored maps of pixel intensity representing activated GB119 at 35 minutes are shown on the right. (**b**) The averages of total signal/area/volume from the slices for each animal is plotted over time for normal (n = 3) and tumor (n = 6) groups. The groups did not differ at time 0 (p = 0.65). Using Bonferroni correction with overall significance level 0.05, the groups differed significantly at times 5, 15, 30 and 35 minutes. (**c**) Inhibition of GB119 activation in an orthotopic brain tumor model using opposing brain slices (opened like a book) containing tumor (arrow, n = 11). (**d**) Percent inhibition of GB119 activity was determined for each mouse and is presented as mean +/− standard deviation at 35 minutes. Using Bonferroni correction for multiple testing at times the groups did not differ at time 0 (p = 0.86) but differed significantly at times 1, 15, 30 and 35 minutes (p<0.0003). Scale bar, 5 mm.

Next, we assessed the ability of the probes to discriminate tumor tissue from normal brain tissue in a mouse orthotopic brain tumor model. For these studies mice were unilaterally stereotactically implanted with Gli36Δ5EGFR cells (**Fig. 3a**, top panel). When tumors were 10–12 days old, animals were sacrificed, the brains removed and sectioned into 2 mm thick coronal sections. Only the brain sections containing visible tumor (arrow, **Fig. 3a**) were used in these studies (3–5 slices/brain, total of 20 slices). Age matched normal mouse brains or sham implanted brains were used as controls for background activation of the probe (**Fig. 3a**, lower panel). After obtaining base-line fluorescent image of the slices, we topically applied GB119 to both hemispheres of the brain slice. The tumor-containing hemisphere of brain consistently and rapidly activated the probe, and activation was always observed at the tumor-brain interface. Neither the contralateral hemisphere nor the normal matched control slices activated GB119 (**Fig. 3a,b**). The slices were washed after 30 minutes and re-imaged at 35 minutes to determine the stability of the signal. These manipulations demonstrated that the signal intensity was stable but that background could be further reduced by washing. Again, we confirmed specificity of probe labeling by co-treatment with protease inhibitor and GB119 (**Fig. 3c,d**).

**Figure 4:**
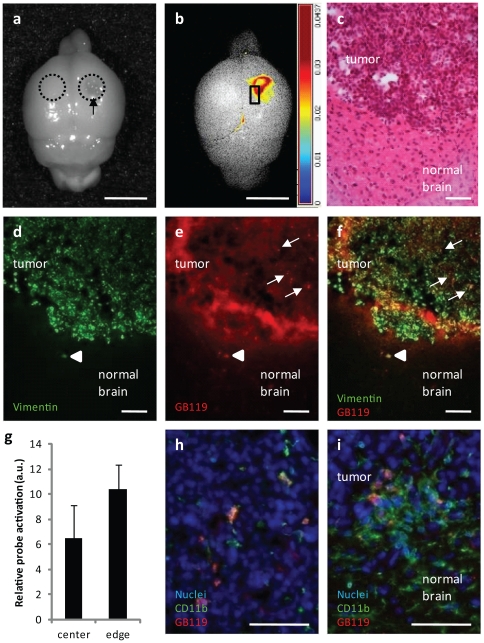
Topical application of GB119 to a brain tumor on the dorsal surface labels the tumor's edge more robustly than the tumor's center. (**a**) Monochromatic image of a whole brain with a tumor growing near the dorsal surface (arrow) showing the treated areas (outlines). (**b**) Unmixed false colored map of pixel intensity representing activated GB119 at 35 minutes post application. (**c**) Horizontal H&E stained section showing higher magnification of square area in panel b demonstrating tumor mass and normal surrounding brain. (**d**) Adjacent sections revealing human vimentin positive cells labeling tumor edge and center (false colored green), (**e**) activated GB119 (Cy5, false colored red) revealing infiltrating cells, the tumor margin and to a lesser degree the interior of the tumor and (**f**) merged imaged demonstrating co-registration (yellow) of GB119-labeled cells and vimentin positive cells within the tumor mass (arrows) and outside of the main tumor mass, arrow head. (**g**) GB119 labeled cells at the tumor's edge more robustly than at the tumor's center, although not significantly, p = 0.124. (**h** and **i**) reveal that GB119 is associated with CD11b-positive cells, but only when they are tumor associated, (**h**) is a typical image observed from within the tumor interior and (**i**) shows the images typical of the tumor – brain interface. Note that only CD11b positive cells associated with normal brain do not activate the probe. Section thickness 25 µm (**d–f**) and 10 µm (**h**,**i**). Scale bar, 5 µm (**a**,**b**), and 100 µm (**c–f, h,i**).

For a subset of the tumor-bearing brains where the tumor was visible on the dorsal surface of the brain no sectioning was performed (n = 6). Instead we removed the brain and applied the probe directly to the tumor and surrounding tissue, and as a control, probe was also applied to a similar sized area on the contralateral hemisphere (**Fig. 4a**, outlines). Only probe application to the tumor side resulted in signal, which was detectable by 5 minutes and continued to increase over time (**Fig. 4b**). Again, activation was largely localized to the tumor-brain interface (**Fig. 4b**). To further resolve which tissue was associated with activated probe, the brains were fixed in paraformaldehyde and subjected them to immunohistochemistry and fluorescence microscopy. These studies showed the majority of activated probe (false colored red) was associated with vimentin-positive human tumor cells (false colored green), with the greatest extent of activation being present at the tumor margin (**Fig. 4e,f**) and associated with either human or mouse Cat L expression (**figure S2**). The Cy5 fluorescence of the probe at the margins, however, appeared more diffuse than the punctate staining noted within the tumor mass itself, and may be the result of activation of secreted proteases into the interstitial space at the tumor margin as well as an effect of epifluorescence resulting from GB119-labeled cells in different planes within the 25 micron thick sections used in this particular experiment (**Fig. 4e**, arrows). Notably, GB119 was able to recognize a vimentin-positive cell outside of the main tumor mass (**Fig. 4f**, arrowhead), i.e. infiltrating tumor cells. Using Image J we were able to quantify the fluorescence from cells located within the tumor center or at its edge. These data suggested more probe activation occurred in cells located at the edge of the tumor but did not reach a statistical significance, p = 0.124 (**Fig. 4g**). Interestingly, immunostaining of these sections for a microglial and macrophage marker, CD11b, suggested that some but not all of the tumor-associated signal resulted from microglial activation of the probe (note co-registration of microglial and GB119 Cy5 signals (**Fig. 4h**). Microglial activation of probe only occurred in microglial cells that were tumor associated. High concentrations of microglial cells in normal brain adjacent to the tumor did not activate the probe (**Fig. 4i**).

**Figure 5:**
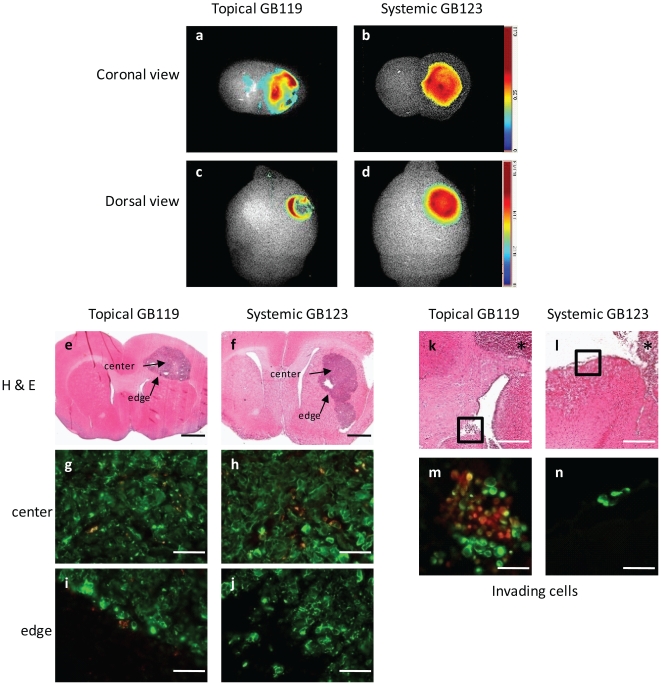
Comparison of tumor cell labeling between topical application of GB119 and systemic administration of GB123. (**a–d**) Representative unmixed false colored maps of pixel intensity of coronal 2 mm sections (**a**,**b**) and dorsal surface tumors (**c**,**d**) comparing the pattern of activation of topical GB119 (left side) and systemic GB123 (right side). (**e,f**) H&E coronal sections demonstrating tumor mass. (**g–j**) Merged fluorescent microscopy of adjacent sections showing labeling in the center (**g,h**) and edge (**I,j**) of the tumor. Sections were immunostained with anti-human vimentin antibody to reveal the brain tumor xenograft (false colored green), covalently bound Cy5 (false colored red). Yellow represents co-localized staining. (**k & i**) H&E sections demonstrating tumor cells that migrated away from the main tumor mass (outlined) via the ventricular system (**k**) or along the cortical meninges (**i**). Boxed regions indicate invading cells (**m** & **n**). Merged images of boxed regions from panels k & i showing labeling of invading tumor cells. Vimentin positive tumor cells (false colored green) and Cy5 cells (false colored red) reveals that invading cells were identified by topical GB119 (yellow cells) (**m**) but not by systemic GB123 (**n**). DAPI staining for cellular orientation was also included in panels **m,n**. Scale bar, 1 mm (**e,f**), 500 µm (**k,l**), and 50 µm (**g–j, m, n**).

The tumor-brain interface associated staining by GB119 (**Fig. 3** **and** **4**) was in contrast to other studies where IV injection of GB137 and GB123 showed signal uniformly throughout flank tumors [31]. To determine if the route of administration or the implantation site altered the pattern of staining we directly compared imaging of orthotopic brain tumors using systemically administered GB123 or topically administered GB119 (GB119 is not stable for IV injection [23], [31]). Both probes were able to define the tumor, but the pattern of Cy5 fluorescence was not identical (**Fig. 5**). In both whole brain and brain slice models, low resolution imaging using the Maestro system suggested that systemically administered GB123 probe appeared to uniformly label the entire tumor mass (**Fig. 5b,d**). This was in contrast to the edge labeling detected when GB119 probe was topically applied (**Fig. 5a,c**). Histologic evaluation of sections from these tumors indicated that systemic administration of the probe primarily labeled cells within the interstitial spaces of the tumor mass (**Fig. 5h**) while not highlighting the tumor edge (**Fig. 5j**). In contrast, tissues probed topically with GB119 showed punctate staining within the tumor mass, primarily associated with tumor cells (**Fig. 5g**), and reproducibly highlighted the peri-tumor space and tumor cells and CD11^+^ cells within that space (**Fig. 5i**).

To more precisely represent the signal intensity between GB119 and GB123 we quantified the probe fluorescence in microscopic sections using Image J. Quantification and statistical comparison of the signal measured from the center of tumors labeled with topical GB119 or from the center of tumors labeled with systemically administered GB123 revealed that there was no statistical difference between the signal intensity. In contrast, the signal resulting from the edge of GB119 treated tumors was 12-fold greater than that coming from GB123 treated tumors and did reach statistical significance, p>0.05. Remarkably, in a subset of animals where some tumor cells had moved throughout the brain, presumably migrating along either the ventricular system or the meninges, topically administered GB119 robustly identified these cells and clusters (**Fig. 5k,m**), while systemically administered GB123 did not in other animals (**Fig. 5l,n**).

**Figure 6:**
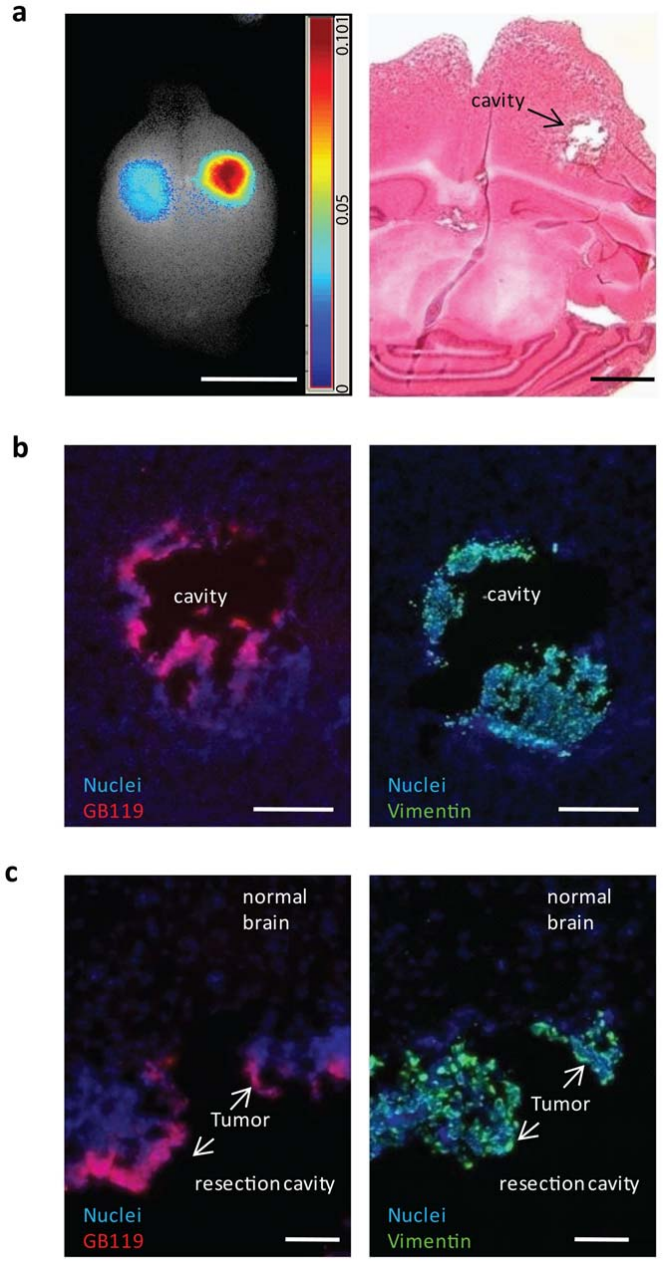
Topical probe administration reveals remaining tumor tissue in incomplete tumor resection. (**a**) Gross resection of tumor growing on dorsal surface of brain was performed and GB119 was added directly to resulting cavity and imaged over time. H&E stained section demonstrating the resulting tumor cavity with remaining tumor cells (**right**). (**b**) Adjacent sections demonstrating GB119-labeled tissue (false-colored red, **left**) is associated with vimentin-positive tumor xenograft (false-colored green, **right**) but not with normal brain tissue. (**c**) Higher magnification of cavity edge from (b). Cell nuclei are labeled with DAPI (false-colored blue) in (**b and c**). Section thickness 10 µm. Scale bar, 5 mm (**a, left**) 1 mm (**a, right**), 500 µm (**b**), 100 µm (**c**).

We also sought to determine if topical application of probe to surgically resected tumors could be utilized to identify tumor tissues that were “missed” during the resection. In animals where the tumor grew to the dorsal surface of the brain the tumors were removed and the resection cavity was filled with GB119. This resulted in activation of GB119 (**Fig. 6a**) in each case (N = 5). The brains were then sectioned and the fluorescing tissue identified (**Fig. 6b and c**). Only remaining tumor tissue fluoresced, while normal brain was devoid of signal. As is evident from **Fig. 6**, topical application of the probe only results in a partial penetration into tumor tissue.

**Figure 7:**
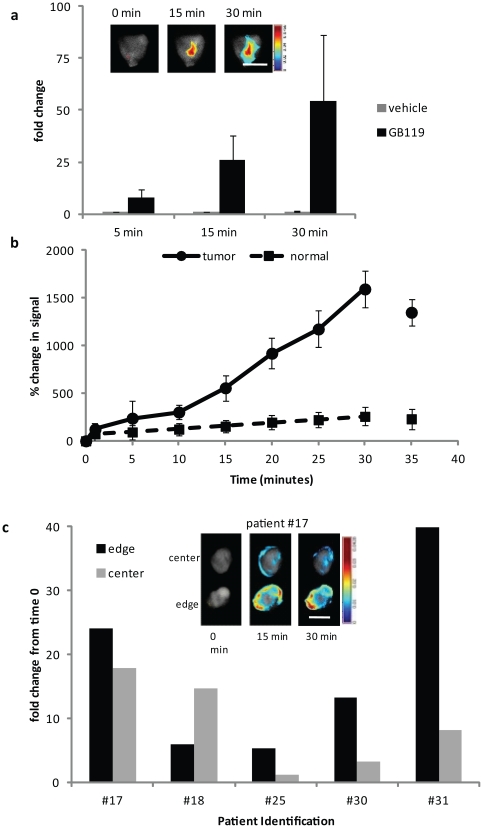
Rapid *ex vivo* activation of GB119 in human GBM biopsies. (**a**) Fold change in total TSAV across time for tumor biopsy pieces compared to vehicle treatment alone (n = 34). 14/15 of patients tested showed probe activation in at least one piece but only 34/42 (81%) of pieces tested had activation greater than 4-fold that measured in normal human brain tissue. Inset is a representative unmixed false colored map of pixel intensity representing activated GB119 in a tumor piece imaged over time. (**b**) Percent change in total signal of 3 pieces of normal brain frontal lobe compared to 3 pieces of tumor treated with GB119 from a single patient. Data are plotted as mean +/− SD across time. (**c**) Left, representative images from a single patient showing background and unmixed false colored maps of pixel intensity representing activated GB119. Right panel is fold change in total signal/area/volume at 30 minutes for edge and center biopsy samples for 5 different patients. Scale bar, 5 mm.

The final step in these studies was to demonstrate that topically administered GB119 could also be activated by GBM tissue derived from human patients. Discarded human GBM tissue was obtained from the operating room and treated with GB119 within 30 minutes of resection. Robust activation of the probe was measured in 14 out of 15 patients in as little as 5 minutes. Activation continued for at least 30 minutes, although varying degrees of activation was measured among different tumor pieces (**Fig. 7a**). In a single surgical case, resection of the tumor mass also provided samples of non-tumor human brain, or “normal brain”. Probing of this patient's tumor and matched “normal brain” tissue demonstrated a significant activation of GB119 by tumor tissue with little activation by the “normal” brain tissue (**Fig. 7b**). Since the results from our orthotopic model (**figure S2**) and reports in the literature indicate that Cat L protease expression/activity is upregulated at the tumor margins, we examined directed biopsies for probe activation. Patient tissue samples from both the edge and center of the tumor, as defined by the surgeon, were obtained from five patients and imaged over time as previously described. Within this set of samples, eight out of ten rapidly activated the probe, with greater activation in the edge samples from four of the five patients. The two samples that did not show activation were both biopsies from the center of the tumor (**Fig. 7c**). These data suggest that there is heterogeneity of protease expression both within and between tumors. However this finding did not reach statistical significance with our limited sampling.

## Discussion

Currently, there is a significant need for new imaging tools that can be used to define tumor margins during the process of surgical resection. While many new approaches have been developed that make use of various affinity reagents and enzyme substrates and have demonstrated utility in animal models, only one targeted probe has been demonstrated for real-time intra-operative molecular imaging applications in humans [28], [32]. Here we demonstrate an alternative application of fluorescently quenched activity based probes, which can be used topically to identify tumor tissues. These reagents are sufficiently small and hydrophobic so that they can be applied topically and freely penetrate into associated tissues. In addition, because the probes are able to covalently label their target proteases, it is possible to use direct biochemical methods to confirm the specificity of probe labeling. In this study we demonstrate the utility of this approach for visualization and potential better resection of human brain tumors, but envision an expanded set of applications in other tumor systems that express high levels of cysteine cathepsin activity.

Glioblastoma multiforme is one of the most lethal human cancers. Technological advances in brain surgery have significantly impacted patient survival and quality of life [10], [12], [33]. To further advance image guided brain tumor resection, several groups are now pursuing fluorescent dyes or drugs (especially photodynamic therapy agents) to label tumors. Attempts to improve tumor resections include applications of non-targeted fluorescent probes [34], [35], [36], targeted agents, such as quantum dots [37] or labeled peptides [38] and substrate based imaging probes [39], [40], [41]. Most recently a first in human trial has demonstrated the feasibility of a targeted imaging agent to elaborate disseminated ovarian cancer in real-time during surgery [28]. However, these trials still have to demonstrate an impact on patient survival.

A trial investigating orally administered 5-ALA, a photodynamic therapeutic, to guide surgical resection of brain tumors has demonstrated that fluorescence-guided resections result in better brain tumor removal with an increase in MRI-negative post-operative scans and an increase in disease free survival at 6 months [3]. However, delivery of 5-ALA into brain tissue is non-uniform because it depends on a leaky blood brain barrier. This combined with a sometimes weak fluorescent signal from tumor tissues and/or high fluorescent backgrounds, can confound identification of tumor tissue during surgery. Furthermore, 5-ALA patients remain photosensitive after administration.

GB119 is a hydrophobic small molecule quenched optical imaging probe that produces virtually no fluorescence signals in tissues that do not express active cathepsin proteases. This characteristic allows for topical application and results in rapid and robust identification of even very small amounts of tissue-associated protease activity. We present data demonstrating that topical application of GB119 can rapidly identify peri-tumor spaces and margins, *including cells that were not detectable by systemic delivery of a similar but not identical un-quenched probe, GB123*. The technology presented solves many of the problems faced by systemically administered probes (e.g. 5-ALA and FITC–labeled probes). Namely, 1) GB119 is labeled with a Cy5 dye that fluoresces in the near-infrared region of the spectrum, potentially eliminating much of the auto-fluorescence background encountered with probes in the visible wavelength such as FITC; 2) GB119 can be directly and uniformly applied to unidentifiable tissue during the surgical procedure and is immediately bioavailable, even to tissues with poor vascularization, elaborating tumor tissues from normal tissues in 5–10 minutes; 3) Topical administration appears to be more effective than systemic administration for identification of small clumps of tumor cells.

Our studies demonstrate that the pattern of probe activation is dependent on the mode of administration. With systemically administered agents the pattern of probe activation was uniform and limited to the tumor mass. In contrast, topical administration of probe consistently produced an activation pattern that was primarily associated with the peri-tumor space.

Immunohistochemical studies revealed that within the tumor mass punctate labeling of cells co-staining for tumor markers were much more prevalent with topical application of GB119 than with systemically administered GB123. In contrast, most of the labeling within the tumor mass for GB123 was associated with cells that were negative for human tumor tissue markers and located in apparent interstitial spaces within the tumor (**Fig. 5**). Of note CD-11b positively staining cells also activated probe but only when associated with cancer tissues. In each case, activation of GB119 was associated with regions of high Cat L expression, **figure S2**. Cat L is regulated by endogenous inhibitors. Since the antibody recognizes active as well as inactive Cat L, these data show that Cat L while distributed within the tumor and normal brain is differentially regulated and active within the brain and tumor-brain interface only.

In several studies GB119 activation occurred on the contralateral side of the brain. In all of these examples H&E staining of tissue sections demonstrated “satellite” colonies of tumor cells on the contralateral side of mouse brains unilaterally implanted with tumors. However, the satellites cells were not labeled in animals that received systemic application of GB123. From these observations we hypothesize that infiltrating tumor cells are not well vascularized and therefore proteases in these regions are refractory to labeling by systemically administered probes. Although generally these satellites would not be exposed during human surgery, this data does suggest that topically administered GB119 might be able to better identify tumor cells at the margin that have not yet generated a vascular system then systemically delivered imaging agents.

Another finding unique to the topical application of GB119 was the robust tumor-margin labeling. Further, the labeling of the peri-tumor space with GB119 (largely absent with GB123) was notably diffuse. The diffuse labeling and definition of tumor margins after topical application of GB119 may also be a result of secretion of activated proteases into the interstitial space at the tumor margin and due to the thicker sections used to obtain signal.

We also tested the utility of topical application to identify “missed tumor” in a mouse model for tumor resection. These studies clearly demonstrated that topical application of GB119 following tumor resection could easily be used to identify tumor tissue remaining in the resection cavity. It is of some concern that in resection of tumors in a living animal blood and other fluids entering the resection field might interfere with imaging. Our flank tumor studies in living animals in which cavities were surgically prepared within the tumors and imaged argue strongly that this is not the case. We are currently developing a model for brain tumor surgery in live mice to test this question directly. These studies have also demonstrated that penetration of the probe into tissue is limited. We, therefore, envision this technology to be used to enhance surgical procedures. We envision normal surgical procedures to remove “most” of the tumor mass and the topical application of GB119 to be evoked when there is a question as to the origin of tissue at the edge of the surgical margin. Using an iterative process the surgeon would apply probe, surgically remove fluorescing tissues and reapply the probe, repeating the process until no probe activation is detected. Further, it is possible that this technology may complement 5-ALA or MRI guided surgeries, first cutting to 5-ALA/MRI negativity and then using topical GB119 to identify or “probe” the presence of non-vascularized invading tumor cells. We do not anticipate, although, that these techniques, despite their potential to significantly improve GBM resections, will result in surgical cures for patients. We do, however, believe that the techniques described here can impact patient survival and quality of life [5], [7] and that reduction in tumor burden may potentially impact other non-surgical therapies [13].

Here we explore the concept of topical application of imaging probed to identify cancerous tissue during surgery, which was recently introduced by Kobayashi and co-workers while this manuscript was under review [29]. In this work Urano et al. exploited a cancer-specific cell surface gamma-glutamyltranspeptidase to activate topically applied quenched imaging probe which then became internalized, rapidly and selectively labeling a number of different ovarian cancer lines both *in vitro* and in a mouse model of disseminated ovarian cancer [29]. We demonstrate that a NIRF quenched activity based imaging probe, GB119, that targets tumor proteases can be used to differentiate tumor cells from surrounding normal brain tissues. Tissue labeling with fluorescently quenched activity based probes for the cysteine cathepsins is highly protease selective and associates with peri-tumor space and brain tumor margins, consistent with over-expression of these proteases in these locations (**figure S2** and [21], [22]). In studies utilizing human GBM tissue biopsies we demonstrated that this technology is robust and has the potential to be rapidly applied in the clinical setting. Taken together these data strongly suggest that topical application of an optical imaging probe directly into the surgical field is a feasible way to visualize tumor tissues. Topical application of optical imaging agents should improve cancer cell identification during surgery, enable near real-time feedback on the completeness of the surgical resection and therefore warrants further development.

Both topical and systemic administration of molecular imaging probes to elaborate tumor tissues have demonstrated their potential utility in mouse models with a human clinical trial now underway to assess image-guided ovarian cancer surgery with systemically administered probes. A driving force for acceptance of intraoperative molecular probes into surgical use will be the development of adequate imaging hardware technology, that will allow the seamless translation and use of molecular imaging probes in the surgical workflow [42], [43], [44], [45], [46]. As a field we should be working towards a set of technical requirements both for agent development and hardware development to allow for rapid development and maximum utility of these agents and hardware so cross platform use of agents and devices is possible.

## Materials and Methods

### Ethics Statement

All procedures were performed aseptically according to the Case Western Reserve University Institutional Animal Care and Use Committee (Case IACUC). The work performed in this manuscript was approved by the Case IACUC; CWRU IACUC Animal Experimental Protocol: 2009-0019.

### Tissue culture, in vitro and ex vivo labeling

Human Gli36Δ5EGRF glioblastoma cell lines obtained as a gift from Dr. EA Chiocca [47] were grown in DMEM (4.5 g/l glucose, L-glutamine) supplemented with 10% fetal bovine serum and 180 U/ml penicillin, 180 µg/ml streptomycin, 0.45 µg/ml amphotericin B (all reagents from Gibco, Invitrogen Corporation). These cells constitutively over-express the vIII mutant forms of the *egfr* gene and were selected for with the addition of 10 µg/ml of puromycin. Cells were grown at 37°C in a 5% CO_2_ atmosphere (Thermo Forma). For labeling experiments Gli36Δ5EGFR cells were incubated with a 50 µM JPM-OEt, a general papain-family inhibitor or 0.1% DMSO as a vehicle control. One hour later 1 µM of the indicated probe (GB119, GB123, or GB125) was added for 4 hours. Samples were analyzed by SDS-PAGE and Cy5 fluorescence was measured by scanning the gels with a Typhoon fluorescent scanner (GE Healthcare). *Ex vivo* analysis of labeling in Gli36Δ5EGFR flank tumor tissues was done in 10–12 day old tumors removed from mice. The tissue was divided into pieces and each immersed in 10 µM GB119 for the 5, 10, 15, 20, 25, or 30 min or in 100% DMSO vehicle control for 30 minutes. Additionally Gli36Δ5EGFR flank tumor was incubated for 15 minutes in either vehicle only (DMSO), GB111-NH_2_, an inhibitor of cathepsins B and L (inhibitor) [23], cathepsin L probe only (GB119), or GB119 probe plus a 100-fold excess of GB111-NH_2_ inhibitor (P+I). Post incubations samples were washed in saline and analyzed by SDS-PAGE as above.

### Cell Culture Preparation for Heterotopic and Orthotopic Implants

Cell harvesting for brain and flank implantation was performed after washing the cells in PBS and a short incubation with 1 ml 0.05% trypsin-EDTA (Gibco, Grand Islands, NY). Trypsin was inactivated by the addition of serum-containing media and the resulting cell suspension was centrifuged at 1000× *g* for 3 minutes. Cells were re-suspended in 10 ml PBS, counted and washed 2 times in PBS. The cells were re-suspended in 2 µl PBS/mouse for brain implants and 250 µl PBS∶matrigel (BD Biosciences) for flank tumor implants per animal (3×10^5^ cells per animal for brain implants and 3×10^6^ cells for flank tumor implants). Immediately following the cell harvesting procedure animals were inoculated.

### Animal Surgery

Athymic nude female mice (*nu/nu*, 6–8 weeks at time of surgery) were bred and maintained at the Animal Resource Center at Case Western Reserve University according to institutional policies (CWRU IACUC Animal Experimental Protocol: 2009-0019). For brain tumor implants, mice were anesthetized by intraperitoneal injection of 50 mg/kg ketamine/xylazine and fitted into a stereotaxic rodent frame (David Kopf Instruments, Tujunga, CA). A small incision was made just lateral to midline to expose the bregma suture. A small (1.0 mm) burr hole was drilled at AP = +1, ML = −2.5 from bregma. Glioblastoma cells (for preparation see above) were slowly deposited at a rate of 1 µl/minute in the right striatum at a depth of −3 mm from dura with a 10 µl Hamilton syringe (26G blunt needle, Fisher Scientific). The needle was slowly withdrawn and the incision was closed with 2–3 sutures. For flank tumor implants, mice were anesthetized with inhaled isofluorane∶oxygen for immobilization. The matrigel∶cell mixture (for preparation see above) was loaded into a 1 ml syringe fitted with a 26-gauge needle and kept on ice. The mixture was injected subcutaneously in the right flank region of the mouse. The needle was withdrawn and the animal was returned to the cage.

### Immunostaining and histology

Post imaging brains were fixed in 4% paraformaldehyde, 30% sucrose protected and frozen in optimum cutting temperature compound (OCT) for cryosectioning (Leica CM3050S). Sections were collected serially at 10 or 25 µm cut directly on to slides and stored at −80C until processing. For probe visualization slides were warmed to room temperature for 10 minutes, washed in PBS and coverslipped. Immunostaining was carried out after washes in PBS with triton X 100 (PBSTX), blocking in appropriate normal serum and incubation in primary antibody for 1–2 hours at room temperature. For visualization of human tumor tissue, a human anti-vimentin antibody (NeoMarkers) was used at 1∶1000. Fluorescent secondary antibody was labeled with Alexa 488 (Molecular Probes) so as to not interfere with the visualization of the Cy5-labeled probe. For visualization of human and mouse cathepsin L, Anti-human Cathepsin L antibody (RD Systems Cat#: AF952) and Anti-mouse Cathepsin L antibody (RD Systems Cat#: af1515) were used. All fluorescent sections were counterstained with the nuclear dye DAPI (Sigma-Aldrich) and coverslipped with fluorescent mounting media (Fluor-Gel, Electron Microscopy Sciences). Hematoxylin and eosin staining was carried out on adjacent sections by standard procedures.

### Microscopy

Fluorescent images were viewed with a Leica DM4000B upright microscope (Filter cubes A4 bandpass (BP) 360/470, L5 BP 480/527, and Y5 BP 620/700 for excitation and suppression filters, respectively) and captured with a QImaging Retiga EXi Fast 1394 camera with a Mercury 100W bulb (CHIU Technical Corporation) as the light source. Camera and software (QImaging Q Capture Pro version 5.1.1.14) were powered by a Dell Optiplex GX620 desktop computer. Equal exposures and magnification were used for documentation. Images were documented in 12-bit gray scale and converted to false color jpeg images. Merging of images taken with different fluorescent cubes was carried out in Adobe Photoshop CS3 Extended version 10.0.1 by first converting the original tif image to 8-bit, adjusting gray scale levels equally, and merging channels to assign appropriate false colors. Image J (NIH software) analysis of Cy5 fluorescence was carried out using the original image, assigning equal threshold values for the DAPI and the Cy5 images, then analyzing the area of the fluorescent images. For each image, the Cy5 area was divided by the DAPI area to obtain percent Cy5 fluorescence. Three mice were used for the analysis taking values from a representative image of the tumor center, edge, or invading cells.

### Imaging and analysis

*In vivo* imaging of heterotopic flank tumors was performed under inhaled isofluorane∶oxygen. The skin overlying the tumor and the tumor capsule was removed and 10 µM GB119 was topically applied in 2–3 µl drops with a pipet tip directly on the tumor tissue. The mice were euthanized immediately post imaging with Fatal Plus. *Ex vivo* imaging was performed after euthanasia of the mice and removal of the brain. The brain was either kept intact or sectioned in 2 mm slices using an acrylic 1 mm coronal brain matrix (Kent Scientific) prior to application of 4 µl of GB119/hemisphere for the slice data. In studies where brains remained intact 2 µl/site on tumor and normal brain were applied. Lateral tail vein systemic GB123 injections (0.5 µl of a 50 mM solution, 33.3 µl DMSO and 66.6 µl PBS/mouse) were performed 19–20 hours prior to imaging. The human tumor biopsies were collected under a University Hospitals Case Medical Center approved IRB protocol (#12-05-17). Tissue was collected in sterile saline and pre-imaged followed by topical application of 3–10 µl drops of 1 or 10 µM GB119 within 30 minutes of biopsy excision. Fresh biopsies were obtained from 15 patients (1–3 pieces/patient; total of 42 pieces). There was no difference in the change in activation when 1 or 10 µM applications of probe were compared. Spectral fluorescence images were obtained using the Maestro™*In-Vivo* Imaging System (CRi, Inc., Woburn, MA). A band-pass filter appropriate for the Cy5 (yellow filter set, Ex 575–605 nm, Em 645; acquisition settings 630–850) was used for emission and excitation light, respectively. The tunable filter was automatically stepped in 10 nm increments while the camera captured images at a constant exposure of 1000 ms. Fluorescence images were acquired before treatment, immediately post treatment and every five minutes for 30 minutes. At this time the treated areas were washed with 100 µl of saline to remove any unbound probe and imaged again at 35 minutes.

To compare signal intensities, regions of interest (ROI) were selected over the entire treated area (tumor or non-tumor) and the change in fluorescence signal over baseline was determined. To do this baseline fluorescence was measured, the probe was applied and the subsequent images were acquired. The spectral fluorescent images consisting of autofluorescence spectra and activated imaging probe were captured and unmixed based on their spectral patterns using commercially available software (Maestro™ software 2.4.0, Cri, Woburn MA). Total signal in the ROI in photons measured at the surface of the animal was divided by the area the probe was applied to (in pixels) then divided by volume of the probe in order to compare activation between animals and imaging experiments where different amounts of probe were administered. Spectral libraries were generated by assigning spectral peaks to the tissue background fluorescence (either *ex vivo* tissue or mouse whole body), background from the imaging stage, unactivated quenched probe applied to the skin, and activated probe over tumor at maximal activation. The spectral libraries were manually computed using the Maestro™ software, with each mouse or tissue used as their own background control. A spectral shift was found between the unactivated probe and activated probe (**figure S3 and S4**). This difference allowed us to differentiate between activated and unactivated probe and all analysis and quantitation of activated probe was based on the spectral library determined from this shift.

### Statistics

Coefficients of variation (CV's) were first determined for total signal (TS), total signal/volume of probe (TSV), total signal/area probe was applied to (TSA), and total signal/volume/area (TSVA). TS and TSV tended to have higher CV's than TSA or TSAV, indicating that correcting for area helps reduce inherent between-animal variability. For all analysis the measure of TSVA was used. For the flank tumor data, the Wilcoxon signed rank test was used to test whether the signal at each of the time points 1, 5, 15, 30 and 35 differed from the signal at time 0. For the orthotopic brain slice, dorsal surface, and human biopsy data, to better meet assumptions of normality underlying the statistical methods, the logarithms of total signal/area/volume were used in the analysis. These data were analyzed using a mixed linear repeated measures model, and means of control and treated were compared at each timepoint, with Bonferroni correction for multiple testing at times >0. Data are presented as means and standard deviation on the original untransformed scale.

## Supporting Information

Figure S1 **Cat L labeling resulting from** ***in vivo*** **topical application of GB119 to exposed flank tumors.** *In vivo* labeling of Gli36D5 flank tumors with 5 mM (lane 1) or 1 mM (lane 3) GB119 or no treatment (lanes 2, 4). Flank tumors were treated topically with probe and the red areas were dissected out and analyzed by SDS-PAGE as in **figure 1**. Specific Cathepsin L bands are indicated as follows; Cat L sc. Minimal tissues could be resected and therefore only the abundantly labeled Cat L sc was visible.
(TIF)

Figure S2 **Immunohistochemical analysis of probe specificity for cathepsin L protein following topical application to explanted brain.** Twenty-five µm sections were used to visualize covalently bound GB119 in (**a**) the tumor center (**b**) the tumor edge, and (**c**) the contralateral hemisphere (columns labeled Tumor center, Tumor edge, and Contralateral hemisphere). Immunostaining was performed to identify either (**d**) human cathepsin L in the xenograft tumor or (**e**) mouse cathepsin L in the host brain at the tumor edge, or (**f**) in the contralateral hemisphere. Panels (**g–i**) are DAPI counterstained sections to visualize cell nuclei/density. Panels (**j–l**) are merged images of GB119, cathepsin L protein and Dapi for tumor center, tumor edge and contralateral side, respectively. These data reveal yellow cells both at the center of the tumor and at the tumor's edge suggesting GB119 covalently binds to active cathepsin L enzyme both within and around the tumor, but not in the normal brain surrounding the tumor nor in the contralateral hemisphere. These data also suggest that mouse tissues, likely macrophages, contribute to the Cat L activity at the tumors edge, also see figure 4 in the manuscript. Scale bar = 100 µm.
(TIF)

Figure S3 **Determination of spectral library for analysis of activated GB119.** (**a**) Monochromatic image showing heterotopic Gli36D5EGFR flank tumor and tube containing 5 ml of 10 mM GB119 (arrow) (**b**) Unmixed spectral library from mouse body (white line), imaging stage (black line) both chosen from the before treatment image. The blue line is unactivated GB119 chosen from both the tube (arrow) and probe applied to skin (arrow head) in **c** immediately after probe application. The green line was chosen from probe over tumor at 30 minutes (arrow) in **d**. Images in **c** and **d** are from the raw data in the image cube. A measurable shift in the spectral profile of the activated (green line) compared to unactivated (blue line) probe was observed.
(TIF)

Figure S4 **Use of spectral library to determine activation of GB119.** (a) Spectral library showing mouse background (white line), imaging stage background (black line), activated GB119 subtracted from mouse background (red line), and unactivated GB119 subtracted from mouse background (magenta line). Library derived as in figure S3 above. (b) Image is the unmixed composite showing the false-colored hotmap of the activated probe (left side) or the unactivated probe (right side) over the background image at 30 min. (c) Time course of total signal of activated GB119 over time in both tumor (solid line) and skin (dotted line). Spectral profiles derived here were used to analyze all *in vivo* and *ex vivo* imaging experiments.
(TIF)
