# Supplementary figures and images for: Topical Application of Activity-based Probes for Visualization of Brain Tumor Tissue
Source: PLoS One. 2012 Mar 13;7(3):e33060. doi: 10.1371/journal.pone.0033060 (PMC3302795; doi:10.1371/journal.pone.0033060)

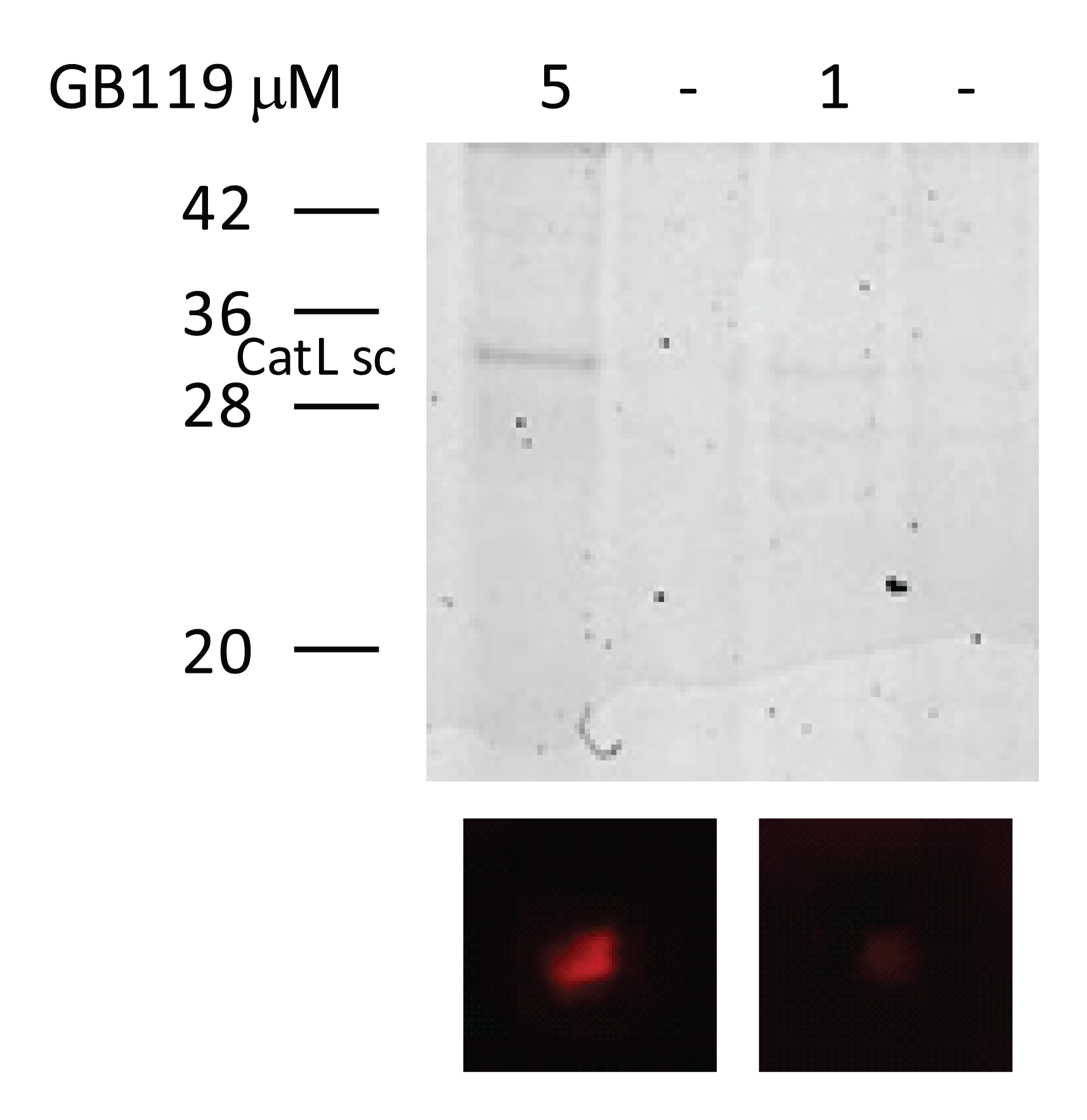

Supplement: Figure S1 — Cat L labeling resulting from in vivo topical application of GB119 to exposed flank tumors. In vivo labeling of Gli36D5 flank tumors with 5 mM (lane 1) or 1 mM (lane 3) GB119 or no treatment (lanes 2, 4). Flank tumors were treated topically with probe and the red areas were dissected out and analyzed by SDS-PAGE as in figure 1 . Specific Cathepsin L bands are indicated as follows; Cat L sc. Minimal tissues could be resected and therefore only the abundantly labeled Cat L sc was visible. (TIF) [file pone.0033060.s001.tif]

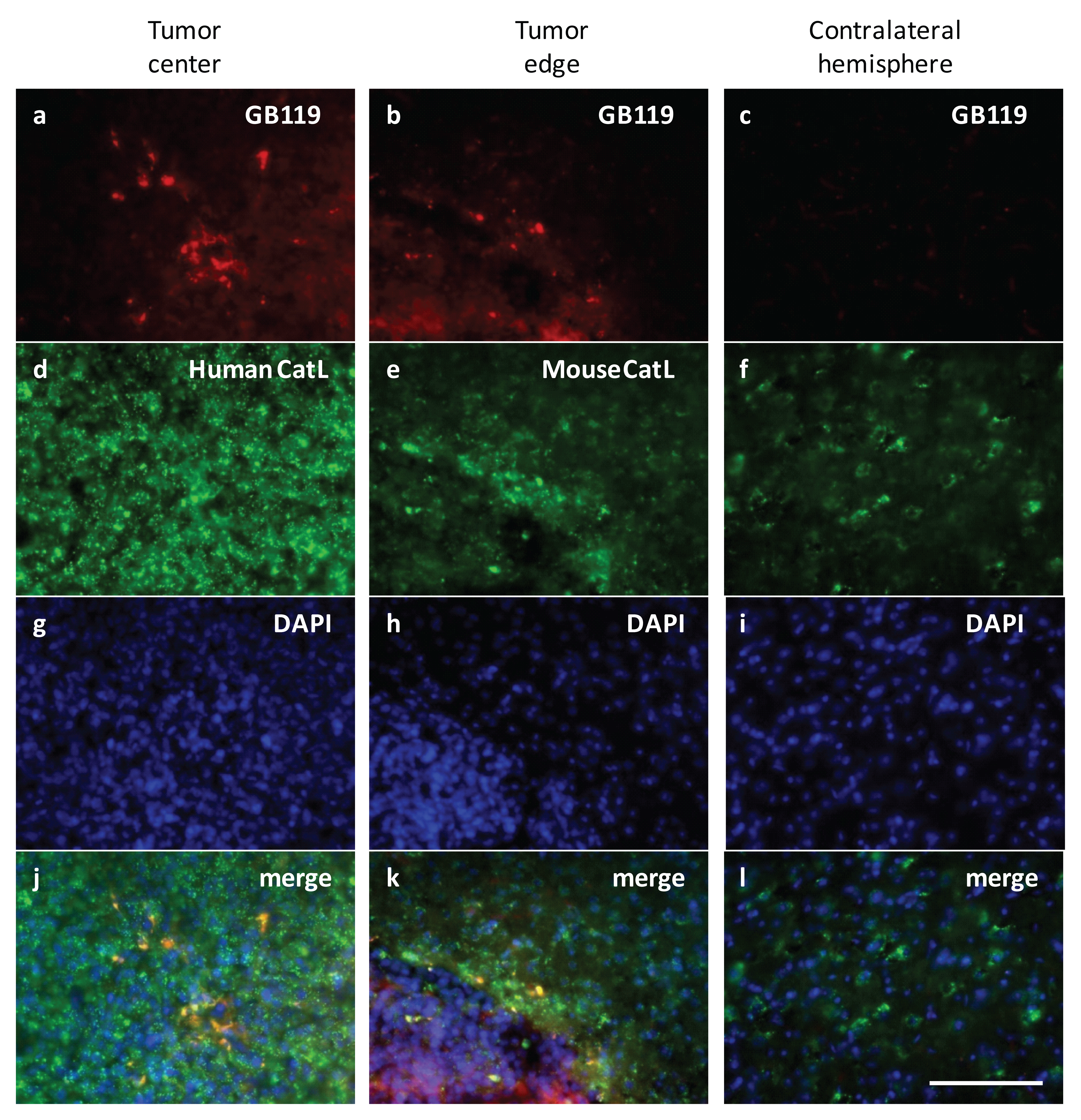

Supplement: Figure S2 — Immunohistochemical analysis of probe specificity for cathepsin L protein following topical application to explanted brain. Twenty-five µm sections were used to visualize covalently bound GB119 in (a) the tumor center (b) the tumor edge, and (c) the contralateral hemisphere (columns labeled Tumor center, Tumor edge, and Contralateral hemisphere). Immunostaining was performed to identify either (d) human cathepsin L in the xenograft tumor or (e) mouse cathepsin L in the host brain at the tumor edge, or (f) in the contralateral hemisphere. Panels (g–i) are DAPI counterstained sections to visualize cell nuclei/density. Panels (j–l) are merged images of GB119, cathepsin L protein and Dapi for tumor center, tumor edge and contralateral side, respectively. These data reveal yellow cells both at the center of the tumor and at the tumor's edge suggesting GB119 covalently binds to active cathepsin L enzyme both within and around the tumor, but not in the normal brain surrounding the tumor nor in the contralateral hemisphere. These data also suggest that mouse tissues, likely macrophages, contribute to the Cat L activity at the tumors edge, also see figure 4 in the manuscript. Scale bar = 100 µm. (TIF) [file pone.0033060.s002.tif]

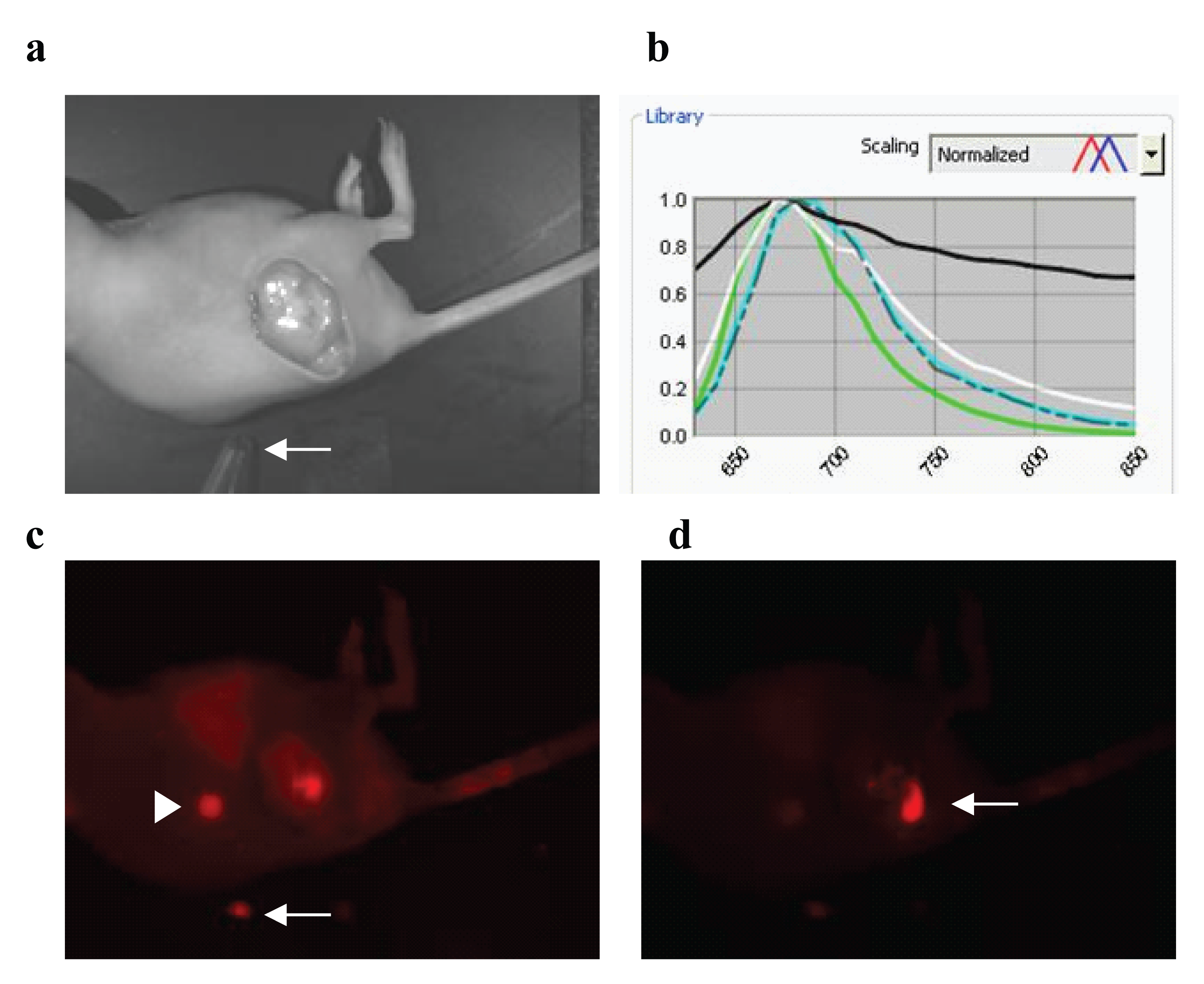

Supplement: Figure S3 — Determination of spectral library for analysis of activated GB119. (a) Monochromatic image showing heterotopic Gli36D5EGFR flank tumor and tube containing 5 ml of 10 mM GB119 (arrow) (b) Unmixed spectral library from mouse body (white line), imaging stage (black line) both chosen from the before treatment image. The blue line is unactivated GB119 chosen from both the tube (arrow) and probe applied to skin (arrow head) in c immediately after probe application. The green line was chosen from probe over tumor at 30 minutes (arrow) in d. Images in c and d are from the raw data in the image cube. A measurable shift in the spectral profile of the activated (green line) compared to unactivated (blue line) probe was observed. (TIF) [file pone.0033060.s003.tif]

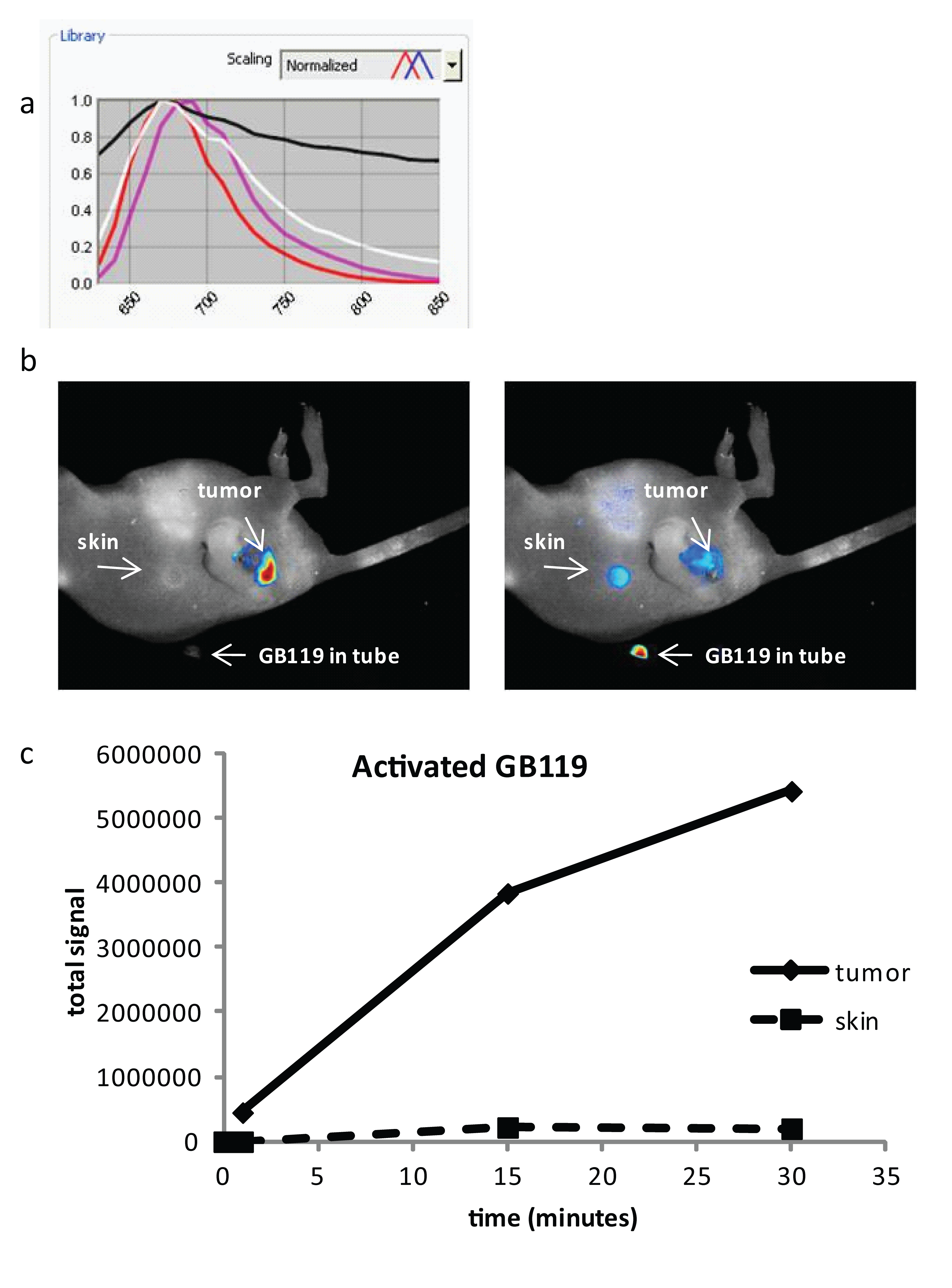

Supplement: Figure S4 — Use of spectral library to determine activation of GB119. (a) Spectral library showing mouse background (white line), imaging stage background (black line), activated GB119 subtracted from mouse background (red line), and unactivated GB119 subtracted from mouse background (magenta line). Library derived as in figure S3 above. (b) Image is the unmixed composite showing the false-colored hotmap of the activated probe (left side) or the unactivated probe (right side) over the background image at 30 min. (c) Time course of total signal of activated GB119 over time in both tumor (solid line) and skin (dotted line). Spectral profiles derived here were used to analyze all in vivo and ex vivo imaging experiments. (TIF) [file pone.0033060.s004.tif]
